# Health-Care-Seeking Patterns in the Emerging Private Sector in Burkina Faso: A Population-Based Study of Urban Adult Residents in Ouagadougou

**DOI:** 10.1371/journal.pone.0097521

**Published:** 2014-05-19

**Authors:** Idrissa Beogo, Chieh-Yu Liu, Yiing-Jenq Chou, Chuan-Yu Chen, Nicole Huang

**Affiliations:** 1 International Health Program, National Yang Ming University, Taipei, Taiwan; 2 École Nationale de Santé Publique, Ouagadougou, Burkina Faso; 3 School of Nursing, National Taipei University of Nursing and Health Sciences, Taipei, Taiwan; 4 Institute of Public Health, School of Medicine, National Yang Ming University, Taipei, Taiwan; 5 Institute of Hospital and Health Care Administration, School of Medicine, National Yang Ming University, Taipei, Taiwan; Iran University of Medical Sciences, Iran (Republic of Islamic)

## Abstract

**Background:**

The private medical care sector is expanding in urban cities in Sub-Saharan Africa (SSA). However, people’s health-care-seeking behaviors in this new landscape remain poorly understood; furthermore, distinguishing between public and private providers and among various types of private providers is critical in this investigation. This study assessed, by type, the healthcare providers urban residents in Burkina Faso visit, and their choice determinants.

**Method:**

We conducted a population-based survey of a representative sample of 1,600 households in Ouagadougou from July to November 2011, consisting of 5,820 adults. We assessed the types of providers people typically sought for severe and non-severe conditions. We applied generalized estimating equations in this study.

**Results:**

Among those surveyed, 97.7% and 53.1% indicated that they seek a formal provider for treating severe and non-severe conditions, respectively. Among the formal provider seekers, 20.5% and 17.0% chose for-profit (FP) providers for treating severe and non-severe conditions, respectively. Insurance coverage was held by 2.0% of those surveyed. Possessing insurance was the strongest predictor for seeking FP, for both severe (odds ratio [OR] = 1.15, 95% confidence interval [CI] = 1.04–1.28), and non-severe conditions (OR = 1.22, 95% CI = 1.07–1.39). Other predictors included being a formal jobholder and holding a higher level education. By contrast, we observed no significant difference in predisposing, enabling, or need characteristics between not-for-profit (NFP) provider seekers and public provider seekers. Proximity was the primary reason for choosing a provider.

**Conclusion:**

The results suggested that FP providers play a crucial role in the urban healthcare market in SSA. Socioeconomic status and insurance status are significant predictors of provider choice. The findings can serve as a crucial reference for policymakers in response to the emergence of FP providers in SSA.

## Introduction

The recent, abrupt growth of the private healthcare sector in countries in Sub-Saharan Africa (SSA) has heightened public interest in investigating changes in behavior of people seeking health care. The private healthcare sector is a sought-after and crucial contributor to the healthcare system, and comparisons between private and public provisions continue to stimulate theoretical and empirical debate [1]–[5]. Earlier studies have predominantly investigated rural settings [6]–[11], but expansion is skewed to urban areas and predicted to thrive [12]. However, only a few studies have focused on health-care-seeking behavior among urbanites in the new healthcare landscape. Because of the availability of numerous therapeutic systems [13], [14], rapid population changes, and rapid urbanization [15], [16], how people living in urban areas seek care might drastically differ from how those living in rural areas seek care [17].

Prospective studies have predicted rapid urbanization in Africa, with approximately 50% growth by 2030 [18]. The population of Ouagadougou City, which exhibited 6.8% growth in 1985 and 7.6% intercensus growth in 2006, is expected to double (1,475,839 million) by 2015 [19]. Incremental and pluralistic private health care combined with the rapid growth of SSA cities defy the aim of health planners to match people's needs and health system capacities [20]. The progressive outlook regarding urban background typically overshadows the negative aspects of health-related challenges experienced by urban center dwellers [21]. Urbanized cities undergo “urban health penalty”, borne health epidemiology inversion, contain numerous health hazards [22]–[25], and endure the “double burden”[26]. Although cities offer a greater number of healthcare institutions [27], care access in the urban context is not characterized by equity. This benefit is provided to affluent minorities [22], [28].

Most previous studies have not distinguished formal sources of care from informal sources of care, which might distort the comparisons between private and public providers. Furthermore, formal private providers are a heterogeneous group. Previous studies have indicated that for-profit (FP) private providers differ substantially from not-for-profit (NFP) providers in incentives, motivation, patient perception, access, and quality of care [17], [29]–[31]. In low- and middle-income countries (LMICs), particularly in SSA, from decolonization until the 1970's, health care and service provisions were state-run [32] and gratuitous [33]. The ideology of the free market has empowered [34], [35] private service providers, who have gained popularity and prominence [36]. In countries such as India, private providers now account for approximately 80% of providers nationwide [27], [37]. In SSA, including Burkina Faso, because of the market liberalization, certain domestic contingencies, such as the sudden regional currency (CFA Franc) devaluation by 50% that occurred in Western and Central Africa in 1994, the Structural Adjustment Program designed by the International Monetary Fund, and the democratization wave of the 1990s, have served as a springboard for the private sector. Private sector development ranks high on the policymaking agenda [38]–[40], and is viewed as an alternative to achieving millennium development goals [38]. This change in the healthcare landscape proves consumer resilience to market-pattern modifications [41], [42].

Health-care-seeking behavior is complex. The Andersen Behavioral Model [43] suggests that using healthcare services involves the predisposing, enabling, and need variables. Predisposing factors, consisting of education and demographic variables, might distinguish the choice between types of care and providers. Hjortsberg [44] also theorized the importance of socio?demographic factors in health-care-seeking behavior. Wong et al. [45] reported that women and older adults preferred public care providers. However, other studies have reported that neither age nor gender affected the preferences of the study sample [46]. The results of previous studies are mixed. Several studies have determined that age, but not gender, is a critical predictor [47], whereas other studies, such as Nuwaha [11], have indicated that men visit private care providers more often than women do.

Previous studies have contended that education influences the choice to visit formal providers, who are most likely private [46], [48], [49]. Numerous results have suggested that income is an enabling factor. Fiedler and Wight [50] posited that the choice of a private provider is income-elastic; in other words, those who are more affluent are more likely to visit private providers [51], [52]. Saksena et al. [53] conducted an ecological study that included only formal providers. In 27 of 39 low-income countries, people in the poorest quintile used public outpatient services, whereas the richest quintile chose the private sector.

The Andersen Andersen Behavioral Model considers insurance an enabling factor. The literature has consistently proven that insurance coverage results in a substantial increase in health system use [54]. One study on dental services in Ouagadougou, Burkina Faso reported that insurance holders sought services at an earlier stage than the uninsured did [55]. In a South African case study, Heever [56] concluded that insurance improves formal health-care provider use. Multiple coverage or enhanced private insurance coverage also increases the likelihood of choosing a private provider [57], [58], even when universal coverage exists [57]. Insurance was indicated to lower the prescription burden [59]. Since the last decade, health insurance coverage has become the primary means advocated by the World Health Organization (WHO) to increase healthcare use and achieve “Health for All” [24].

Finally, regarding need factors, several authors have demonstrated the role of perceived quality of care and perceived severity of condition in choosing private care over other healthcare options [3], [11], [37]. A standard method for categorizing the severity of illnesses is lacking, and authors have used various definitions to describe it. However, acute severe conditions were determined to influence the decision to visit private practitioners [45], and people with less severe diseases also seek private care [46], [60]. In addition to these conditions, people with chronic diseases primarily patronize the public healthcare sector [3].

Develay, Sauerborn, and Diesfeld [61] reported in 1996 that 57.3% and 58.9% of Ouagadougou residents self-treated for mild and slightly serious illnesses, respectively, whereas 54.8% solicited modern care for serious illnesses. However, the healthcare environment has changed dramatically since two decades ago. No studies have documented urbanites’ health-care-seeking behaviors in the current health care environment, with its emerging private sector. Therefore, this population-based study explored people’s choice of provider according to the severity of their conditions in an urban area with an emerging private sector, and further investigated the influencing factors associated with people’s choice of providers. Both formal and informal care sources were analyzed. Among formal providers, three main types of providers (public, FP, and NFP) were also assessed.

## Methods

### Setting

Ouagadougou, similar to other capital cities in Western Africa, is a primary business center and is densely populated [19]. It has the densest health network, and subsequently the largest proportion of health personnel (MD = 39.4%; NP = 33.7%; midwives = 20.8%). The city houses 9.9% of the public health facilities and 60.3% of the private health facilities in Burkina Faso. By 2010, 217 clinical facilities were established in the capital city region, including 7 policlinics, 23 medical centers, 46 medical clinics, and 110 nurse-led clinics [62]. On average, people reside within a mean radius of 1.7 km from a health facility [63]. The nationwide mean radius is 6.4 km.

The healthcare system in Ouagadougou consists of three groups of formal care providers: public, FP, and NFP providers. The public sector consists of three teaching hospitals, four district hospitals, and approximately 60 primary health care centers (PHCs). Most PHCs are headed by nurses, and deliver a comprehensive set of preventive and curative services, essential drugs, and health promotion activities. They also act as gatekeepers for referral services. The public system applies a cost-recovery system based on a user-fee policy [42], [64], and an out-of-pocket scheme is the main financing scheme [65].

Most healthcare facilities registered in Ouagadougou are private providers, including 102 FP providers and 104 NFP providers [66]. The private health sector has rapidly expanded since the market liberalization reforms of 1991, and the number of private providers, particularly FP providers, has increased rapidly, leading to the establishment of the Private Sector Department in 2002 [67]. This government agency oversees the private healthcare sector. Like elsewhere in LMICs [68], the private sector in Burkina Faso was found to encompass a wide range of providers: general and specialized hospitals; clinics led by medical doctors, nurses, and midwives; and laboratory and radiology units.

The present study investigated three main types of formal care providers: public (including frontline facilities, district hospitals, and teaching hospitals), NFP, and FP providers (categorized by the Burkina Faso Ministry of Health). Private providers were defined as all individual-run or group-owned medical institutions that were not managed or owned by the government or a municipality. FP providers were defined as benefit-focused, and NFP providers were defined as philanthropic-oriented medical institutions. Informal practitioners, such as traditional healers, were not considered private (FP/NFP) providers. Only clinical-based care providers were considered in this study.

### Study Population and Sampling Procedure

This study was conducted in Ouagadougou, the capital city of Burkina Faso, which comprises approximately 12.3% of the population of the country [69]. The city has 30 administrative sectors and some attached-villages. The sampling frame consisted of all households within the administrative municipality of Ouagadougou. The attached-villages were excluded. The household was used as the end (secondary) sampling unit. The “common households” such as military barracks, hospitals, and commercial units were also excluded.

In the absence of a household list, we applied the simplified general method for cluster-sample surveys in developing countries [70], [71] developed by the WHO. To maximize the representativeness of the study sample and strengthen its statistical power, we used the cardinal point system for cluster determination and chose South, North, West, and East as the order for all 30 administrative sector (AS) entry points. Two-stage clustered sampling was implemented based on the city map. We first selected streets (the primary sampling unit) in the individual AS using random selection without replacement. From each selected street, starting from the first entry point (South), the right side (of the street) was drawn at random, and a skipped interval applied to single out households to be surveyed (secondary sampling unit). In common plots, only one household was randomly selected. A household was considered a group of people whose food was prepared by the same person [70].

Based on previous studies [17], [46], [49], [72]–[74], we enlisted a representative sample of 1,600 households. To apply the probability-proportional-to-size (PPS) of the AS, the number of households in each of the 30 ASs was obtained from the National Institute of Statistics and Demographics (NISD). We applied a simple three-table formula: 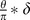, where θ was the total sample size defined (n = 1600 households), and π was the population of households listed by the NISD (n = 277,988). This coefficient was multiplied by the number of households (δ) enumerated in each individual AS.

Of the 1,600 households who consented to participation, 22 households were excluded because they had resided in the city for less than 6 months, or because they failed to complete the interview process. A total of 1,578 households were finally retained. Information on 8,243 individuals was collected, a mean household size of 5.2 persons per household was yielded. The statistic is comparable to the official statistic (5.3 persons per household) reported by the most recent Demographic Health Survey in Burkina Faso [75]. Due to vast differences in morbidities, demand for healthcare, and utilization patterns between adults and children, in this study, we only focused on adults aged 15 years or above in reference to previous studies [47], [76]. There were 5,820 adult individuals in these households.

### Data Collection and Instrument

We developed a structured questionnaire based on a literature review and a set of questionnaires used in the Demographic and Health Survey (DHS), which were developed by the Burkina Faso NISD. Three academics and one field researcher first conducted a content validity check of the candidate questionnaire. Subsequently, forward and backward translations into French were conducted. Finally, the questionnaire was pilot-tested using 32 households from four ASs in the study setting to perform semantic adjustments.

The first section of the questionnaire included questions on household predisposing factors, namely socio?demographic variables, and enabling factors, consisting of people’s occupation and health insurance. The second section consisted of questions regarding provider choice and factors prompted by health conditions (need factors). Finally, 10 questions were asked regarding the reasons for choosing a particular provider (i.e., physical accessibility, financial affordability of health services, and quality of care items).

The interviewees were asked separate questions for their usual choice of provider when encountering severe and non-severe conditions. The question posed to the interviewee was: “Where do you/does a specific individual household member seek care when a severe disease/injury/condition occurs?” A severe condition was further explained by the interviewer as a condition which the patient perceives may result in fatality in the absence of urgent intervention. Examples of common symptoms in Burkina Faso were illustrated as loss of consciousness, coma (generally in a context of a fever), tachypnea, a fracture, or a bad injury caused by an accident. Similarly, for non-severe conditions, the question asked was: “Where do you/does a specific individual household member seek care when a non-severe disease/injury/condition occurs?” Non-severe conditions were further defined by the interviewer as a condition which may compromise everyday activity or work ability if medical care is not sought, but is not critical and is not perceived by the patient as a matter of vital danger. An array of symptoms was listed: headaches, stomachaches, fevers, the shivers, and coughs. These questions were posed to participants to capture their usual tendencies in health care seeking and choice of providers.

In each selected household, the household head and spouse (if any) were personally interviewed in a face-to-face manner. The household heads and their spouses (if any) responded to the survey questions on behalf of all household members. Thus, no children or minors were personally interviewed in this study. To ascertain the validity of the obtained information, the responses of the household head and the household spouse were compared to ensure consistency. The response of the household spouse was retained in the event of conflicting information, under the assumption that spouses are more aware of illness events occurring in the household. Because consumers might use nicknames to designate providers, more detailed provider information was obtained by matching the provider name with the private provider roster published by the Department of Health. We conducted onsite verification because of the presence of unlicensed, new, and unlisted providers. Six interviewers were trained and given a booklet detailing the fieldwork strategy.

Finally, the principal investigator (PI) checked the questionnaire sheets individually for completeness. The data were entered into a mask of the Census and Survey processing package, Version 4.0., and the PI and an independent data entry person double-checked the dataset for discrepancies.

### Study Variables

Two dependent variables were used in this study. First, we assessed provider choice between formal and informal sources of care. Informal sources of care included traditional healers and self-treatment. Second, among those who chose a formal provider, we assessed their choice among public (PHC, district hospital, teaching hospital), FP, or NFP care. The provider choices were analyzed separately for those with severe conditions and non-severe conditions.

Following the Andersen emerging model [43], predisposing factors (age, gender, education, and marital status), enabling factors (occupation and insurance status), and the need for care (perceived illness: severe and non-severe) were included for analyses. Age was categorized into four brackets (15–24, 25–44, 45–64, and 65 years and above). Education level was divided into five levels: university, junior high, junior, primary, and no formal education. Occupation included three categories: formal employment (public and para-public work, and formal private work), informal sector employment, and other, which included those who were retired, household wives, students, and the unemployed. Marital status was defined as being married or single. Because of their small proportion (4.9%), widows and widowers, and those who were divorced or separated, were also classified as single. Regarding insurance status, people were either classified as uninsured or belonged to all other insurance plan categories.

In addition to these characteristics, a set of 10 commonly identified reasons determining provider choice were also assessed (i.e., physical accessibility, financial affordability of health services, quality of care items).

### Statistical Analysis

Because of the hierarchical nature of the household data, the responses of individuals may have been correlated. To manage possible clustering effects, generalized estimating equations (GEEs) were applied [77], in which the first level was individual adults and the second level was households. Because of the programming constraints and according to previous literature, separate GEEs analyses were conducted for NFP providers and FP providers over public providers. In addition of implementing separate GEEs analyses for each type of private provider, we conducted sensitivity analyses with multinomial regression models, to analyze all three types of providers simultaneously. The results remained robust. Data were analyzed quantitatively using SPSS Version 18 and SAS 9.1 packages. Descriptive statistics regarding the reasons that contributed to the choice of provider were also obtained.

### Ethical Statement

Only household heads and their spouses were interviewed in this study. Participants answered questions for themselves and on the behalf of all other household members, including children. No minors or children were interviewed in this study. The informed consent statement, presented on the first page of the questionnaire, was read and explained to all interviewees. Instead of written consent, an oral consent was sought, to maintain a friendly communication environment; a considerable proportion of interviewees in the target population may not have been literate. Furthermore, in prior field experiences, concerns were raised that a written signature would compromise the anonymity of the interview and compromise interviewees’ trusts. The respondents were not therefore asked to sign the form, but were either read the form or allowed to read it themselves, and ticked “yes” or “no” on the consent form accordingly. The verbal consent from the household head and their spouses were documented. When the response was “no,” the interviewers did not attempt to convince them otherwise. Interviewers were asked to politely apologize and leave. All the data used in this study was anonymized. The targeted respondents were clearly informed on the voluntary nature of their participation and could decline their consent at any time. The same strategy is also used in the DHSs. The Burkina Faso National Ethics Committee for Research approved the whole study design including the methods, verbal consent procedure, and the data collection information, #2011–11–82 (11 November 2011) upon the examination and oral presentation of the proposal. Furthermore, administrative permission was obtained from the Ouagadougou Town Council.

## Results

**Figure 1:**
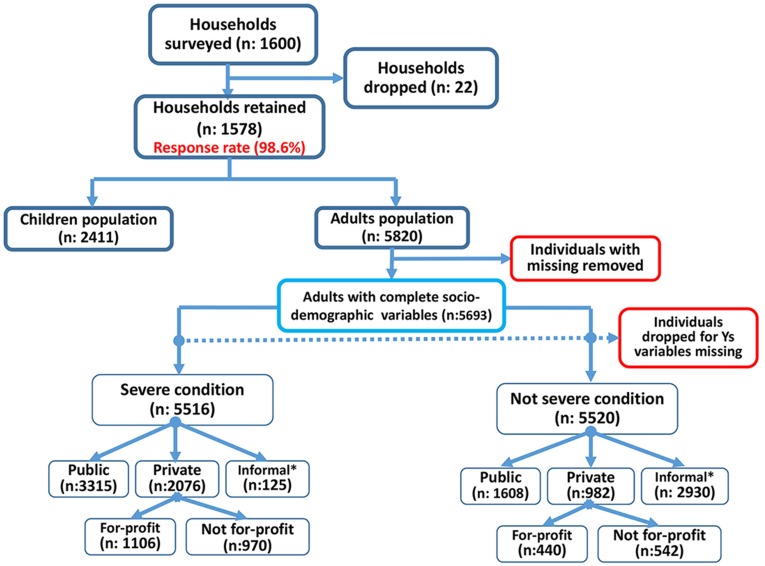
Algorithm of healthcare utilization by urban residents by type of health conditions. *Includes self-treatment, traditional healers and other informal providers.

The overall response rate (i.e., the number of completed and validated interviews divided by the total number of valid contacts) was 98.6%. Of the households interviewed, the information on the choice of provider when encountering severe and non-severe health conditions was reported on the 5,820 adults. Of the 5,820 adults, 127 had missing demographic and socioeconomic status information, and were excluded. Of the remaining 5,693 adults, 177 were missing information regarding their provider choice for a severe condition, and 173 were missing information regarding their provider choice for a non-severe condition, and were excluded. The final analytical samples for severe and non-severe conditions were 5,516 and 5,520, respectively (Figure 1). In dealing with severe conditions, approximately 60.1% (n = 3315) of the participants indicated that they seek public providers and more than one-third (37.6%, n = 2076) indicated that they seek private providers; of this one-third, 53.3% indicated that they seek FP providers. Only 2.3% (n = 125) of the participants indicated that they seek informal sources of care. By contrast, more than half of participants (53.1%) indicated that, in dealing with a non-severe condition, they patronize an informal source of care. Because of the prevalence of informal sources of care, we did not observe any significant variation in critical individual characteristics between people using formal and informal sources of care. Detailed analyses suggested that self-treatment was the salient care behavior (92.8%; results not shown).

Table 1 shows that more than 75% of the sample was aged below 45 years, and the sample included more women than men. Slightly more than 25% of the participants did not have any formal education and only approximately 40% of the sample participants were employed either in the formal or informal sectors. The remaining 60% were dependents. The majority of participants paid out-of-pocket. Only approximately 2% of the participants had health insurance coverage from various sources: private, public, and community-based health insurance.

**Table 1:** Column percentages of sample distribution by characteristics and types of health conditions.

	Type of health conditions (column %)			
Variables	Severe (5,516)		Not severe (5,520)	
	n	%	n	%
**Age** (year)				
15–24	1923	34.9	1925	34.9
25–44	2352	42.6	2352	42.6
44–64	987	17.9	990	17.9
65 &+	254	4.6	253	4.6
**Gender**				
Female	2833	51.4	2839	51.4
Male	2683	48.6	2681	48.6
**Marital Status**				
Married	2652	48.1	2652	48.0
Single/divorced/separated	2864	51.9	2868	52.0
**Education**				
University	674	12.2	677	12.3
Junior high	979	17.7	978	17.7
Junior	1571	28.5	1573	28.5
Primary	788	14.3	790	14.3
No education	1504	27.3	1502	27.2
**Occupation**				
Formal work (Public &Private)	841	15.2	845	15.3
Informal private	1422	25.8	1409	25.5
Other (retired, household wife, student, jobless)	3253	59.0	3266	59.2
**Insurance**				
No Insured	5406	98.0	5412	98.0
Insured	110	2.0	108	2.0
**Formal providers**	**5391**	**97.7**	**2590**	**46.9**
**Informal providers**	**125**	**2.3**	**2930**	**53.1**

Table 2 presents the distribution of provider choices among people who indicated they would seek formal care. On average, 20.5% of the adults chose FP providers in dealing with severe conditions; 17.0% of the participants chose FP providers in dealing with non-severe conditions. The lowest proportion of people who chose FP providers for treating severe conditions were those without a formal education (13.8%), and those employed in informal sectors (15.5%), whereas the lowest proportion of people who chose FP providers for treating non-severe conditions was observed among those without a formal education (13.0%), and those who worked in the informal sector (12.8%). People with a high level of education were more likely to choose FP providers, whereas people without formal education overwhelmingly indicated that they patronize public sector providers (66.9% for severe conditions and 62.1% for non-severe conditions). The most salient results were observed in regard to the possession of insurance. In treating either severe or non-severe conditions, at least 60% of the insured participants preferred FP providers and approximately 22% of the insured participants indicated that they visit government-owned providers; slightly fewer participants indicated that they visit NFP caregivers. Among uninsured participants, the pattern was the opposite.

**Table 2:** Row percentages of people choosing FP, NFP and public providers by sample characteristics and types of health conditions.

	Type of health conditions (row %)							
Variables		Severe (5,391)				Not severe (2,590)		
	n	FP (n = 1106)	NFP (n = 970)	Public (n = 3315)	n	FP (n = 440)	NFP (n = 542)	Public (n = 1608)
**Age** (year)								
15–24	1874	20.7	17.8	61.5	863	16.1	23.8	60.1
25–44	2308	20.5	18.6	60.9	1141	18.6	18.0	63.4
45–64	962	21.0	16.8	62.2	473	14.6	20.9	64.5
65 &+	247	17.0	18.2	64.8	113	17.7	28.3	54.0
**Gender**								
Female	2772	21.0	17.8	61.2	1361	17.8	20.9	61.3
Male	2619	20.0	18.2	61.8	1229	16.1	21.0	62.9
**Marital status**								
Married	2104	21.5	19.0	59.5	1309	17.8	20.1	62.1
Single/divorced/widow	2787	19.5	17.1	63.4	1281	16.1	21.8	62.1
**Education**								
University	654	32.1	16.1	51.8	346	25.5	17.6	56.9
Junior high	958	25.4	17.8	56.8	512	18.7	21.1	60.2
Junior	1528	20.4	16.8	62.8	746	15.8	19.6	64.6
Primary	770	17.7	19.7	62.6	316	16.1	19.0	64.9
No education	1481	13.8	19.3	66.9	670	13.0	24.9	62.1
**Occupation**								
Formal work (Public & Private)	818	31.8	18.3	49.9	468	25.0	18.2	56.8
Informal private	1392	15.5	18.6	65.9	568	12.8	25.2	62.0
Other (retired, household wife, student, jobless)	3181	19.8	17.6	62.6	1554	16.1	20.2	63.7
**Insurance**								
Insured	107	63.5	15.0	21.5	64	59.4	18.7	21.9
Not insured	5284	19.6	18.1	62.3	2526	15.9	21.0	63.1

**Abbreviations**: FP, for-profit; NFP, not for-profit.

As shown in Table 3, among those who patronized a formal provider, GEE models were applied to identify characteristics associated with their choice of FP, NFP, or public provider. For treating either severe or non-severe conditions, women were slightly more likely to choose FP providers than men were, although the confidence interval (CI) exhibited a marginal significance. For treating severe conditions, participants with a junior high (odds ratio [OR] = 1.03, 95% CI = 1.01–1.04) or university level education (OR = 1.04, 95% CI = 1.02–1.07) were significantly more likely to choose FP providers than were those without any formal education. Enabling variables, including occupation and insurance, were observed to be significantly associated with visiting FP providers. In addition, people working in formal sectors were significantly more inclined to choose an FP provider (OR = 1.03, 95% CI = 1.01–1.04). After adjusting for other factors and clustering effects, insurance was the strongest predictor among the participants for seeking care from FP providers, for treating either severe (OR = 1.15, 95% CI = 1.04–1.28) or non-severe (OR = 1.22, 95% CI = 1.07–1.39) conditions. Because of the low prevalence of insurance coverage (2.0%), the adjusted ORs may be different from the crude ORs. By contrast, we observed no significant difference in individual characteristics between those who chose public and NFP providers. Similar provider choice patterns were observed for treating non-severe conditions.

**Table 3:** GEE results for provider choices by health conditions among urban residents in Ouagadougou.

	Severe				Not severe			
Variables	For-profit		Not for-profit		For-profit		Not for-profit	
	OR 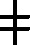	95% CI	OR 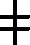	95% CI	OR 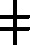	95% CI	OR 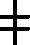	95% CI
**Age,** year								
15–24	1		1		1		1	
25–44	0.99	0.97–1.01	0.97	0.95–1.00	1.00	0.99–1.00	1.00	1.00–1.00
44–64	1.00	0.96–1.03	0.97	0.93–1.01	0.99	0.98–1.01	1.00	1.00–1.00
65yo &+	0.98	0.93–1.03	1.00	0.94–1.06	1.01	0.99–1.03	1.00	1.00–1.00
**Gender**								
Male	1		1		1		1	
Female	1.01	1.00–1.02	1.00	0.99–1.01	1.01	1.00–1.01	1.00	1.00–1.00
**Marital status**								
Single/divorced/widow	1		1		1		1	
Married	1.01	0.98–1.04	1.02	0.98–1.07	1.00	0.99–1.01	1.00	1.00–1.00
**Education**								
No education	1		1		1		1	
University	1.04	1.02–1.07	1.00	0.96–1.04	1.03	1.01–1.04	1.00	1.00–1.00
Junior high	1.03	1.01–1.04	0.98	0.95–1.01	1.01	1.00–1.02	1.00	1.00–1.00
Junior	1.00	0.99–1.02	0.98	0.96–1.01	1.00	1.00–1.01	1.00	1.00–1.00
Primary	1.02	1.00–1.03	1.00	0.97–1.02	1.00	0.99–1.02	1.00	1.00–1.00
**Occupation**								
Other (retired, household wife, student, jobless)	1		1		1		1	
Formal (Public & private)	1.03	1.01–1.04	1.02	0.98–1.05	1.03	1.01–1.04	1.00	1.00–1.00
Informal private	0.99	0.99–1.00	0.99	0.96–1.01	0.99	0.98–1.00	1.00	1.00–1.00
**Insurance**								
Not Insured	1		1		1		1	
Insured	1.15	1.04–1.28	1.02	0.99–1.05	1.22	1.07–1.39	1.00	1.00–1.00

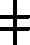
OR (95% confidence intervals) is derived by GEE analysis. All the estimates are adjusted for each covariate for the effects.

of the other covariates. Public provider is used as reference group against FP, and NFP provider choice.

**Abbreviations**: GEE, generalized estimating equations; FP, for-profit; NFP, not for-profit; OR, odd ratio; CI, confidence interval.

We investigated an array of reasons that prompted provider-seeking (Table 4). The proximity to the facility and the competence of the provider were the primary reasons for people to choose a provider, regardless of the provider’s type and the severity of the condition. However, whereas promptness distinguished people with either severe or non-severe conditions who sought FP providers, it was not among the primary reasons for choosing NFP or public providers for treating non-severe conditions. Rather, the cost of the service provision was a major factor. For those seeking care from public providers, low cost was the main reason they chose a public provider.

**Table 4:** Column percentages of sample distribution by reasons prompting provider choice and types of health conditions.

	Type of health conditions (column %)					
	Severe			Not severe		
*Variables*	FPn = 1105	NFPn = 970	Public n = 3310	FP n = 428	NFPn = 539	Public n = 1607
24 h/day services	5.1	6.7	2.8	3.3	3.1	0.7
Closeness	31.1	48.1	47.4	51.9	58.1	77.8
Promptness	19.4	12.4	5.2	8.9	5.8	0.8
Competence	31.0	25.0	28.1	22.0	15.6	3.0
Good drug	2.2	0.4	1.7	0.2	0.0	0.6
Good material	2.2	1.4	1.8	0.0	0.0	0.0
Prior satisfaction	2.3	2.8	1.4	1.6	2.4	0.5
Cheapness	1.6	1.9	7.5	5.1	11.9	15.2
Connection	4.2	0.5	2.6	4.9	3.1	1.1
Other factors	0.9	0.8	1.5	2.1	0.0	0.3

**Abbreviations**: FP, for-profit; NFP, not for-profit.

## Discussion

This study features four main findings. First, urbanites in Burkina Faso predominantly indicated that they use formal sources of care for treating severe conditions. However, for non-severe conditions, more than half indicated that they use informal sources of care or self-treatment. Second, among those who indicated that they seek formal sources of care, approximately 38% sought private providers, both FP and NFP. This study confirmed that private providers constitute a considerable share of the market in urban Burkina Faso. Compared with NFP providers, FP providers constituted a greater share of the formal sector (20.5% for people with severe conditions) and a nearly equal share (17.0% for people with non-severe conditions) of the market. FP providers play a more critical role in treating more severe conditions. Therefore, the role of the private sector, particularly FP providers, in health care systems should not be overlooked. A more comprehensive understanding and additional research efforts are crucial to policymaking.

Third, enabling factors, such as the possession of insurance, exerted a greater influence on provider choice than predisposing factors (including demographics or education) did. After adjusting for other covariates, the insured were observed to be 15% more likely than the uninsured to choose FP providers. This estimate might be modest. It is consistent with the findings of previous studies that having insurance improves formal healthcare use and increases the likelihood of choosing a private provider [56], [78], [79]. Because FP services are typically expensive, having insurance might facilitate the removal of financial barriers in seeking FP care [57], [58]. In Burkina Faso, few people have these benefits because no universal insurance coverage exists. Holding insurance is an indicator of high socioeconomic status or wealth that offers the opportunity to seek private, essentially FP, providers.

Consistent with the results of previous studies [80], [81], education and occupation were significantly associated with FP-provider seeking, although the magnitudes were small. We offer two possible explanations for this result. First, few previous studies collected information regarding insurance status. Controlling for insurance status may have lessened the influence of factors such as education or occupation, which is what we observed in this study. Second, because of the nested structure of our household survey, we applied GEEs to perform logistic regression; multinomial logistic regression was commonly used in previous studies, and GEEs yield smaller estimates than multinomial logistic regression does.

Fourth, whereas a clear difference was observed between FP and public providers, no differences in individual characteristics were observed between those who chose NFP and public providers across the assessed conditions. In Burkina Faso, NFP care institutions date back to the colonial period and are led by various organizations (religious missions, churches, Protestant organizations, Muslim organizations, civil society organizations, and nongovernmental organizations). Although NFP providers are distinguished from public providers in the type of care organizations they comprise, and in stewardship, they share several crucial similarities, including pricing policy –the lowest in the market– and comprehensive packages that include public health programs. They are also granted public employees whose salaries are paid by the government. These commonalities might explain the similar characteristics of people choosing NFP and public providers. The results also demonstrate the importance of clearly distinguishing between FP and NFP providers in healthcare research and health policymaking.

Another observation in this study was the marked difference in the reasons for choosing certain types of providers. Consistent with the findings of previous studies, provider proximity was the primary reason behind people’s choice of providers, even in an urban setting with a high density of health facility networks. Provider competence also ranked high in determining people’s choice of providers for those with either severe or non-severe conditions. The provision of inexpensive services was also a crucial reason marking the difference between choosing either a public or private provider, and was one of the three most cited reasons by public provider-seekers with either severe or non-severe conditions. The incentive of inexpensive services also replaced promptness in importance for NFP provider-seekers treating non-severe conditions. FP providers are considered more responsive, communicative, and more convenient in operating hours compared with public providers [82]. Evidence regarding the promptness of FP providers in the present study for both the assessed conditions concurs with Kin et al.’s study [83], that found that public sector providers are crowded, and that patients experience long waiting times, encounter rude behavior according to Berlan [84], and are required to pay informal fees, for Liaropoulos et al. [85]. Gauthier and Wane [86] conducted a study in Chad and contended that affluent people bypass public and religious clinics and search for providers that provide quality services, despite the 3.5-times higher cost of private clinics.

A few limitations of this study should be noted. First, this study was a cross-sectional study. Therefore, causal relations were not ascertainable. Second, the distance to various types of providers was not included in the analyses, which might have influenced the results. However, we believe that this influence may be small because healthcare providers are densely located in the capital city. The difference in distance to each type of provider from individual households might not be as large as that in rural areas. Third, data on certain crucial enabling factors, such as income or wealth, were not available. Based on prior field experience and the questionnaire pretest, people were often reluctant to answer questions regarding income or wealth. Therefore, to maintain a high participation rate and to collect accurate information, sensitive questions regarding income or wealth were not included, and we collected information on other crucial socioeconomic indicators, such as education, occupation, and insurance status. Fourth, several possible confounding factors, such as provider quality and perception of provider quality, were not assessed in this analysis. Future researches with more comprehensive data may help to contribute in this regard. Finally, this study was conducted in the capital city of Burkina Faso, a typical urban city in SSA with an emerging private healthcare sector. Therefore, the findings may not be generalizable to suburban or rural areas.

### Conclusion and Implications for Policy and Research

This study is one of the first population-based investigations of medical-care-seeking-behavior in an urban setting to address the recent emergence of the private sector in SSA. We analyzed the health-care-seeking behavior of urban residents and observed that the private sector, particularly FP providers, constitutes a considerable share of the market. Two points are worth emphasizing. First, our results indicated that seeking care from FP providers was highly correlated with enabling factors, particularly insurance coverage. In the context of Burkina Faso, our findings clearly demonstrate how insurance coverage facilitated choosing FP providers, who were perceived to be differentially prompt and competent. That the insured chose FP providers in greater numbers emphasizes the role of quality. Extending insurance coverage not only increases people’s health care utilization, but may also provide people with more care options. This may help to narrow the disparities in the quality of care people receive, by allowing people to seek care from more competent providers they need. These results may clarify policy discussions on the realization of universal insurance coverage in developing countries. Second, we suggest that policymakers consider private providers an essential aspect of the formal healthcare system, and pursue greater efforts toward public and private partnership. Achieving public-private partnerships through a contracting system, leasing, or social marketing are possible methods [5], [87], [88]. The Department of Private sector of the Burkina Faso Ministry of Health should develop the capacity to oversee and strengthen management support for an enhanced information system. An independent evaluation reported that a comprehensive assessment of private sector contributions is required [89]. The findings of this study can serve as an essential reference for policymakers regarding the mounting role of private providers in SSA countries, such as Burkina Faso.

### What is Known Regarding this Topic:

- The private sector in LMICs is thriving, and most recently in SSA.
- Complementary health insurance (in the national coverage context), a higher level of education, and a high income are associated with the choice of private provider.
- Acute health conditions influence the choice of private providers, whereas people with chronic conditions favor public providers.
- Compared with public providers, private providers are more responsive in providing services.

### What this Study Contributes:

- This study confirms the existence of a considerable market share of private providers in urban Burkina Faso, and further demonstrates the large presence of FP providers in the healthcare market.
- Insurance coverage, a higher level of education, and employment in the formal sector are crucial predictors of seeking care from FP providers, but not from NFP providers.
- No significant difference in predisposing, enabling, and need factors was observed between people seeking care from NFP providers and public providers.
- Inexpensive service provision distinguishes public providers from private providers in treating severe conditions, and distinguishes public and NFP providers from NF providers in treating non-severe conditions.
